# Targeted fetal NGS panel reveals genetic conditions in sonographically normal fetuses: Insights from a large cohort study

**DOI:** 10.1371/journal.pone.0336979

**Published:** 2025-12-02

**Authors:** Han-Ying Chen, Yi-Ting Wang, Jessica Kang, Yi-Yun Tai, Ti-Jia Yuan, Shin-Yu Lin, Shi-Ming Lin, Chien-Nan Lee, Tsang-Ming Ko

**Affiliations:** 1 Program for Precision Health and Intelligent Medicine, Graduate School of Advanced Technology, National Taiwan University, Taipei, Taiwan; 2 Department of Obstetrics and Gynecology, National Taiwan University Hospital, Taipei, Taiwan; 3 Institute of Molecular Medicine Genetic Counseling Program, College of Medicine, National Taiwan University, Taipei, Taiwan; 4 Department of Medical Genetics, National Taiwan University Hospital, Taipei, Taiwan; 5 Department of Obstetrics and Gynecology, National Taiwan University Hospital Yunlin Branch, Douliu, Taiwan; 6 Genephile Bioscience Laboratory, Ko’s Obstetrics and Gynecology, Taipei, Taiwan; 7 Institute of Medical Device and Imaging, National Taiwan University, Taipei, Taiwan; Shaheed Rajaei Cardiovascular Medical and Research Center: Rajaie Cardiovascular Medical and Research Center, IRAN, ISLAMIC REPUBLIC OF

## Abstract

Prenatal genetic testing plays a crucial role in prenatal diagnosis and is traditionally performed when abnormalities are detected on fetal ultrasound. With the widespread availability of next-generation sequencing (NGS), genetic screening is increasingly being applied to fetuses with normal ultrasound findings. This study aimed to evaluate the diagnostic yield and outcomes of NGS panel testing in a large cohort of pregnant women with sonographically normal fetuses. A retrospective analysis was conducted on 1,820 sonographically normal fetuses that underwent fetal NGS-targeted panel testing based on parental requests between June 2021 and June 2023. Among the 1,820 cases analyzed, no pathogenic mutations were identified in 833 (45.8%), 893 (49.1%) had an abnormal carrier status, and 94 (5.2%) exhibited pathogenic conditions, including 25 autosomal dominant, 29 autosomal recessive, 39 X-linked (35 hemizygous glucose-6-phosphate dehydrogenase [*G6PD*] cases), and one mitochondrial disorder. The most common autosomal recessive mutation was a homozygous pathogenic variant of *GJB2* (19 cases). Furthermore, 48 patients carried heterozygous *G6PD* mutations, and 344 patients were identified as carriers of *GJB2* variants. Other notable findings included 15 cases of familial hypercholesterolemia, five cases of Noonan syndrome, and two cases of osteogenesis imperfecta. The rare disorders identified were Wilson’s disease, cystic fibrosis, Cockayne syndrome, and ototoxic hearing loss, all of which were observed in a single case. A fetal NGS-targeted panel yielded critical findings in 5.16% of sonographically normal fetuses, emphasizing its potential use in prenatal diagnosis. Effective screening requires careful variant selection and detailed pre- and post-test genetic counseling to ensure the clinical relevance and informed decision-making of parents.

## Introduction

Prenatal genetic diagnosis is one of the most significant scientific advances in this field. It provides parents with an opportunity to understand the affected gene and its associated diseases, enabling them to make appropriate responses and preparations. Prenatal genetic testing includes targeted tests and genomic approaches at different resolutions, such as karyotyping, chromosomal microarray analysis, whole-exome sequencing, and whole-genome sequencing. Targeted approaches include QF-PCR and gene panel analyses.

The introduction of next-generation sequencing (NGS) in recent years has transformed the landscape of genetic diagnostics by enabling high-resolution, comprehensive, and efficient variant detection. Studies have reported that more than half of the cases with anomalies remain undiagnosed after karyotyping and chromosomal microarray analysis [1,2]. The International Society of Prenatal Diagnosis recommends karyotype and chromosomal microarray analysis for all pregnant individuals, whereas exome sequencing should be reserved only for cases in which fetal anomalies are present [3]. However, certain ultrasonographic findings are subtle or even undetectable, particularly in cases of metabolic disease, and thus, may be overlooked. With the decreasing cost and turnaround time (TAT) of exome sequencing (ES), it is likely that in the near future, some women will request exome sequencing of their apparently healthy fetuses, or at a minimum, screening for monogenic disorders.

As technological advances and sequencing costs have decreased, some pregnant patients have begun to seek a more comprehensive understanding of their babies’ health. In clinical practice, it is becoming increasingly common to encounter women who require more detailed testing beyond chromosomal microarray analysis. While studies have been conducted on prenatal whole-ES of sonographically normal fetuses, this approach presents significant challenges [4,5], as it cannot detect all genetic variants and may yield misleading conclusions for parents. Additionally, the lack of fetal phenotypic information complicates the analysis of genomic data and makes it challenging to provide accurate genetic counseling.

Studies have proposed that a new list of prenatally actionable findings should be offered alongside the ACMG secondary findings list [6]. This list should include disorders for which fetal therapies are available, such as Pompe’s disease, other lysosomal storage disorders, and cobalaminopathies. Furthermore, disorders with severe, treatable, and early-onset phenotypes not included in the recommended Uniform Screening Panel should also be considered.

In this context, ES has emerged as a valuable approach because it enables comprehensive variant detection without prior knowledge of the gene–phenotype relationship. ES is well accepted as a useful tool in pediatric [7] and adult genetics [8], as it does not require knowledge of the gene–phenotype relationship; moreover, its application has now extended to prenatal diagnosis. Several guidelines now support the use of NGS for prenatal genetic diagnosis [9]. The diagnostic yield rate reportedly ranges from 6% [10] to 89% [11], depending on factors such as cohort size, inclusion criteria, and interpretation of results. A meta-analysis reported a pooled yield rate of 31%, underscoring its clinical utility in patients with abnormal structural findings [12].

Ongoing studies are evaluating the implementation of exome-based gene panel screening in newborn screening programs. However, rather than deferring testing until delivery, our study aimed to facilitate prenatal diagnosis. As our facility receives referrals for genetic testing from both geneticists and obstetricians for a variety of indications, we evaluated the diagnostic yields of exome-based gene panel testing using NGS at our prenatal diagnosis care center.

## Methods

We conducted a retrospective chart review of the genetic results of 1,820 cases between June 2021 and June 2023. Institutional committee approval for data access was obtained, starting on 06/05/2024, for research purposes. All cases were referred to our facility by geneticists or obstetricians in Taiwan for various indications, including advanced maternal age and a history of a previously affected sibling. Importantly, all patients had normal prenatal ultrasound findings before sampling.

Fetal DNA was obtained from chorionic villus samples, amniocentesis, or cord blood. Peripheral blood was collected from both parents for trio-based confirmation to determine whether the detected variants were inherited or de novo. DNA extraction from amniotic fluid and chorionic villi was performed using the gSYNC™ DNA Extraction Kit (Geneaid Biotech Ltd., New Taipei City, Taiwan). DNA extraction from cord or parental blood samples was performed using the MagCore^®^ Genomic DNA Tissue Kit (Code No. 401; RBC Bioscience Corp., New Taipei City, Taiwan).

The sequencing workflow included four major steps: (1) sample library preparation, (2) sample library amplification, (3) sequencing, and (4) data analysis. Targeted libraries were prepared using Agilent SureSelect gene capture technology (Agilent Technologies, Inc., Santa Clara, CA, USA), followed by sequencing on the Illumina MiSeq and Illumina NextSeq2000 platforms (Illumina, Inc., San Diego, CA, USA). The average read depth was 150 × , with 98% of the bases achieving ≥20 × coverage. The reference genome was the human genome map GRCh38. Sequence alignment and variant calling were performed using the Burrows–Wheeler Aligner and Genome Analysis Toolkit (BWA–GATK).

The identified genetic variants were interpreted using multiple databases, including ClinVar, dbSNP, gnomAD, Human Gene Mutation Database (HGMD), and relevant literature. This interpretation followed the ACMG guidelines, which focus on classifying variants as pathogenic or likely pathogenic. If a variant was not registered in the aforementioned databases, its potential impact on protein function could be predicted based on the variant type. This information was then compiled to be provided to prenatal physicians as a reference for further testing and clinical decision-making.

### Gene panel

The targeted NGS exome panel used in this study included 390 genes (S1 Table). The gene panel was selected based on the recommended gene list published by the ACMG [13], diseases included in Taiwan’s newborn screening program (e.g., glycogen storage disease type II and adrenoleukodystrophy), common fetal growth abnormalities (e.g., Noonan syndrome and achondroplasia), physical appearance abnormalities (e.g., Kabuki syndrome and congenital familial blepharophimosis), and genes related to connective tissue, musculoskeletal development, metabolic disorders, and neurological disorders.

The selected genes comprised 123 genes with dominant inheritance, 189 with recessive inheritance, 44 with X-linked inheritance, 31 with either dominant or recessive inheritance, 1 mitochondrial inheritance, and 2 others.

Variant nomenclature has been reported in online databases such as ClinVar, DECIPHER, the Database of Structural Variations in the Human Genome (DGV), the HGMD, and Online Mendelian Inheritance in Man (OMIM). The American College of Medical Genetics and Genomics (ACMG) guidelines for Standards and Guidelines for the Interpretation of Sequence Variants classify variants into five categories: pathogenic (P), likely pathogenic (LP), uncertain significance (VUS), likely benign (LB), and benign (B).

All reported cases of pathogenic variants were reviewed by a multidisciplinary team of geneticists, genetic laboratory specialists, and obstetricians.

### Ethics statement

This study was conducted in accordance with the guidelines of the Research Ethics Committee of the National Taiwan University Hospital. Written informed consent for recording clinical characteristics and thalassemia profiles was obtained from all individuals undergoing testing, and the research ethics committee of the National Taiwan University Hospital waived the authorization for the use of de-identified aggregate data (IRB number: 202404061RINB). The committee approved access to the data collected between June 2021 and June 2023 for research purposes from 06/05/2024–05/05/2025.

## Results

A total of 1,820 cases were screened. Of these, 833 showed no anomalies, 893 had an abnormal carrier status, and 94 harbored pathogenic variants. The most frequently observed condition was glucose-6-phosphate dehydrogenase (G6PD) deficiency, followed by *GJB2*-related hearing loss. The detected pathogenic variants are summarized in Table 1.

**Table 1:** Prevalence of positive screening results for pathogenic genes among participants (N = 1,820).

Autosomal chromosomes				
Gene	Disease or condition associated	Case number		Prevalence within whole study group (%)
*GJB2*	Hearing loss	Homozygous patients	19	19.9
		Heterozygous carriers	344	
		Total	363	
*DUOX2*	Congenital hypothyroidism	108		5.9
*HFE*	Hereditary hemochromatosis	67		3.7
*ATP7B*	Wilson’s Disease	60		3.3
*SLC26A4*	Pendred syndrome	51		2.8
*OTOF*	Hearing deficiency	48		2.6
*SLC25A13*	Citrullinemia, type II	43		2.4
*ETFDH*	Glutaric acidemia type IIC	39		2.1
*SLC22A5*	Primary carnitine deficiency	39		2.1
*GAA*	Glycogen storage disease, type II	37		2.0
**X chromosome**				
Gene	Disease or condition associated	Case number		Prevalence within whole study group (%)
		Male Hemizygous	Female Heterozygous	
*G6PDD*	Glucose-6-phosphate dehydrogenase deficiency	35	48	4.6

There were 83 cases of G6PD variants, representing 4.5% (83/1,820) of the cohort. Among them, 57.8% (48 cases) were heterozygous carriers, and 42.2% (35 cases) were hemizygous.

A total of 344 patients had *GJB2* mutations, accounting for 18.9% (344/1820) of the study group. *GJB2*.109G>A was the most common variant, observed in 91.3% (314 cases) of patients with *GJB2* mutations. Among the 19 pathogenic cases, 68.4% (13) were homozygous for the *GJB2*.109G>A p.Val37Ile variant. Other detected conditions included 25 cases of dominant disorders (62.5%), 10 cases of recessive disorders (25%), 4 cases of X-linked disorders (10%), and 1 case of mitochondrial disorder (2.5%).

Familial hypercholesterolemia (FH) was the most common autosomal dominant disorder, accounting for 15 cases. The most common variants detected were APOB: c.10579C > T, p.Arg3527Trp (5 cases); LDLR: c.1721G > A, p.Arg574His (4 cases); and LDLR: c.1747C > T, p.His583Tyr (2 cases). Genes associated with Noonan syndrome were detected in five cases. Two patients refused to confirm the presence of the parental genes. Comparison of the test results with those of the parental genes indicated that two cases had spontaneous mutations, while one inherited the mutation from the mother, who showed no clinical symptoms. Further genetic testing of these family members revealed that the mother’s eldest son carried the same gene and exhibited symptoms of hearing loss, indicating incomplete penetrance of Noonan syndrome in this family.

The third most commonly detected anomaly was a gene related to osteogenesis imperfecta (OI). We identified two gene variants related to OI that accounted for 0.2% of all abnormalities: *COL1A1*: c.2278G > A; c.2308G > A, p.D760N; p.G770S, and *COL1A2*: c.1666G > T, p.Gly556Cys. Other detected autosomal dominant disorders included gene variants associated with tuberous sclerosis and branchio-oto-renal syndrome in two fetuses, respectively.

Among the autosomal recessive disorders, besides the previously mentioned *GJB2*-related conditions, we identified three cases (7.5% of total abnormalities) of congenital hypothyroidism caused by mutations in the *DUOX2* gene. Additionally, two cases (5.0% of the total abnormalities) of citrullinemia were caused by mutations in *SLC25A13*. We also detected one case each (2.5% of total abnormalities) of Wilson’s disease (mutations in *ATP7B*), cystic fibrosis (mutations in *CFTR*), Cockayne syndrome (mutations in *ERCC6*), auditory neuropathy (mutations in *OTOF*), and autosomal recessive polycystic kidney disease (mutations in *PKHD1*).

Among the mitochondrial disorders, we detected one case of ototoxic hearing loss due to an MT-*RNR1* mutation (2.5% of all abnormalities).

## Discussion

This study reviewed fetal NGS tests conducted since 2021, encompassing 1,820 gene interpretation results. Among these, 94 cases were identified as pathogenic or LP variants, spanning conditions that affect hearing, vision, appearance, skeletal structure, metabolism, and overall development. Based on genetic inheritance patterns, 25 cases were classified as dominant disorders, 29 as recessive disorders, 39 as X-linked disorders, and 1 as a mitochondrial disorder. All potentially abnormal cases were followed up with consultations.

The primary goal of prenatal fetal genetic analysis is to enable early detection and preparation, or to provide parents with the ability to make informed medical decisions. Therefore, it is crucial to establish strict screening criteria for selecting and interpreting gene panels to avoid unnecessary concern for families based on test results. When designing a gene panel, it is essential not only to consider common genetic abnormalities in the population and genes associated with newborn screening disorders, but also to account for limitations of genetic detection, such as the presence of pseudogenes. For example, in Taiwan’s newborn screening program, funded by the National Health Insurance, the pathogenic gene for congenital adrenal hyperplasia (*CYP21A2*) has a pseudogene, *CYP21A1P*, which increases the risk of diagnostic error. Because of this risk, *CYP21A2* was excluded from prenatal testing.

In our study, 25 cases were identified as autosomal dominant disorders, with FH being the most common, followed by Noonan syndrome (NS) and skeletal dysplasia (SD). A total of 12 families underwent parental locus confirmation, revealing that 4 cases were de novo mutations, while the remaining 8 cases were inherited from either the father or mother. De novo mutations occurred in both the NS and SD cases, whereas all FH cases were familial.

FH is a late-onset disorder, and its inclusion in prenatal testing is intended to enable early preventive strategies. Identification also supports health surveillance of family members through cardiovascular monitoring and interventions with medication, diet, and exercise. According to *GeneReviews*, FH is most frequently caused by mutations in *LDLR* (>50%) and *APOB* (5–10%). Clinically, FH is defined by markedly elevated low-density lipoprotein cholesterol (LDL-C) concentrations, which increase the risk of cardiovascular disease. A family history is commonly observed, and FH is classified as heterozygous familial hypercholesterolemia (HeFH) or homozygous familial hypercholesterolemia (HoFH), depending on the underlying genetic variation.

In previous genetic analyses of patients clinically diagnosed with FH in Taiwan [5,14], 445 variants were identified, with *LDLR* mutations accounting for 86% (395 cases) and *APOB* mutations for 13% (58 cases). The most frequent variants were *APOB* c.10579C > T (12.6%), *LDLR* c.986G > A (11.5%), and *LDLR* c.1747C > T (10.8%) [15]. Similar findings have been reported in studies of FH-associated genes in Taiwan [14] and their relation to coronary artery disease [16]. Our results corroborate earlier reports identifying *APOB* c.10579C > T as a prevalent variant; however, no patients in our cohort carried the *LDLR* c.986G > A variant. This discrepancy may reflect limited sample size or population differences, resulting in statistical bias.

Previous studies have shown that NS is predominantly caused by de novo mutations; however, in heritable cases, 30–75% of family members carry the same variant [17]. Because of incomplete penetrance [18], clinical manifestations may differ even among members of the same family. The condition can present with prenatal features and may be transmitted to subsequent generations, making it an important target for prenatal screening.

In our study, 29 cases of recessive disorders were identified, the majority being sensorineural hearing loss caused by mutations in *GJB2* (OMIM: 220290). The carrier rate in our cohort was 19.1%. Pathogenic variants in this gene are associated with varying degrees of hearing impairment, a phenotype particularly common in East Asian populations. A previous study reported an allele frequency of 22.92% for *GJB2* in Taiwan, with the *GJB2* c.109G > A, p.Val37Ile variant accounting for 85.9% of cases [19], a finding consistent with our results. Prenatal detection can provide timely psychological support to families, facilitate early referral to otolaryngologists, and enable close monitoring of newborn hearing, the degree of potential impairment, and the optimal timing of intervention.

X-linked disorders not only clarify the health status of the fetus but also provide valuable insights for both male and female family members. In our study, the most prevalent X-linked disorder was *G6PD* deficiency, reflecting its relatively high incidence in the Taiwanese population (1–3%) [20,21]. Previous reports have shown that the most frequent G6PD variants in Taiwan are c.1466G > T (50%), c.1478G > A (21.3%), c.185A > G (7.4%), and c.1114C > T (4.2%) [22], consistent with the distribution observed in our cohort. This condition predominantly affects males, whereas females demonstrate greater phenotypic variability. Newborns with *G6PD* deficiency are susceptible to severe jaundice and may experience hemolysis upon exposure to specific substances. Inclusion of this gene in prenatal testing panels enables early identification and prevention of neonatal jaundice.

Mitochondrial disorders are maternally inherited, and our panel included only the *RNR1* gene, which is associated with ototoxic hearing loss. The primary rationale for testing this gene is to prevent hearing impairment induced by specific medications and to detect familial inheritance. A 2007 study of Taiwanese families with sensorineural hearing loss reported that 3.2% carried the *MTRNR1* m.1555A > G mutation, with additional family members also affected [23]. Only a few patients in that cohort reported aminoglycoside use, underscoring the familial impact of mitochondrial disease and the importance of testing. Such testing enables both the fetus and relatives to recognize their genetic risk and inform healthcare providers before future treatments.

However, these testing methods have inherent limitations. From a technical perspective, NGS may be unreliable for detecting large deletions, amplifications, insertions, inversions, mosaicism, or variants in regions not covered by sequencing, such as untranslated or intronic regions. In such cases, confirmation with gene chip analysis or complementary techniques is required.

The high carrier rate (49.1%) underscores the importance of structured counseling for carrier findings in prenatal NGS panels. All participants received pre-test counseling outlining the purpose, scope, and possible outcomes of testing, with particular emphasis on carrier status. Post-test counseling focused on interpreting carrier results, explaining inheritance patterns, discussing potential implications for the fetus, and presenting reproductive options, thereby facilitating informed parental decision-making and mitigating anxiety associated with carrier findings.

Although this fetal-targeted NGS panel is powerful, it does not capture all variants. Participants were informed that a negative result does not exclude the possibility of a genetic disease. Moreover, the presence of a pathogenic variant does not invariably result in clinical disease, and the absence of such variants does not guarantee that the fetus is unaffected by genetic conditions.

The high detection rates of *GJB2* and *G6PD* variants underscore the clinical value of including these genes in fetal NGS panels. In addition to their prevalence, both conditions have clear and actionable implications. Identification of *GJB2* pathogenic variants enables early referral for hearing evaluation and timely intervention, which are critical for language acquisition and developmental outcomes. Recognition of *G6PD* deficiency informs counseling on the avoidance of oxidative triggers and supports preventive strategies to reduce neonatal jaundice and hemolytic crises. These examples illustrate how the inclusion of relatively common and actionable conditions not only increases diagnostic yield but also delivers direct clinical benefit, thereby reinforcing their prioritization in fetal NGS screening programs.

The inclusion of diverse diseases in fetal NGS panels for phenotypically normal fetuses can improve outcomes by enabling timely intervention, perinatal management, or obstetrical decision-making in pathogenic cases. However, the identification of variants with variable expressivity or incomplete penetrance introduces risks of overdiagnosis, uncertain prognostic interpretation, and heightened parental anxiety. These challenges underscore the need for comprehensive pre- and post-test counseling, transparent communication of result limitations, and the development of guidelines to ensure balanced clinical implementation.

The diagnostic yield of prenatal sequencing varies substantially with cohort selection and panel composition. Han et al. reported very high yields with WES in fetuses with suspected SD, whereas the PAGE study demonstrated an 8.5% yield using a curated 321-variant panel in structurally anomalous fetuses. To the best of our knowledge, this is the first study to demonstrate the feasibility of applying a targeted NGS panel to sonographically normal fetuses, showing that such an approach can reveal monogenic disorders that may otherwise remain undetected in routine prenatal care. This strategy expands the clinical window for early diagnosis and intervention, underscoring the translational value of integrating genomic screening into standard obstetric practice. Although the diagnostic yield observed in this cohort highlights its potential utility, the relatively modest sample size constrains the generalizability of these findings. Future studies in larger and ethnically diverse populations, alongside professional consensus on optimal panel design, are needed to refine clinical application and maximize impact on prenatal management.

## Conclusion

In conclusion, our study revealed that the yield rate of the fetal next-generation sequencing-targeted panel in sonographically normal fetuses was 5.16%. In this era of rapidly expanding genetic information, this panel offers parents who are eager to understand their fetal health an additional option for further examination. Importantly, comprehensive pre- and post-test genetic counseling is crucial when using this targeted panel for sonographically normal fetuses.

## Supporting information

S1 Table List of 390 genes included in the study’s fetal targeted next-generation sequencing panel. (DOCX)
