# Supplementary material for: Targeted fetal NGS panel reveals genetic conditions in sonographically normal fetuses: Insights from a large cohort study
Source: PLoS One. 2025 Dec 2;20(12):e0336979. doi: 10.1371/journal.pone.0336979 (PMC12671786; doi:10.1371/journal.pone.0336979)
Supplement: S1 Table — (DOCX) [file pone.0336979.s001.docx]

Supplementary table 1: List of 390 Genes Included in the Study's Fetal Targeted Next-Generation Sequencing Panel

| ABCA4 | CHM | ERCC8 | HSPG2 | MCOLN1 | PEX5 | SHOX | TREX1 | AR | AMT |
| --- | --- | --- | --- | --- | --- | --- | --- | --- | --- |
| ABCB11 | CHRNG | EVC | IDS | MESP2 | PEX7 | SIX1 | TRIP11 | NR5A1 | GLDC |
| ABCB4 | CHST6 | EVC2 | IDUA | MIR96 | PHOX2B | SIX3 | TRPV4 | COL5A1 | LMX1B |
| ABCC8 | CLCN7 | F12 | IFITM5 | MITF | PKD1 | SIX5 | TSHR | COL5A2 | A2ML1 |
| ABCG5 | CLCNKA | F7 | IFT172 | MMAA | PKD2 | SLC12A1 | TTC21B | PCDH19 | BRAF |
| ABCG8 | CLCNKB | FGD1 | IGF1 | MMAB | PLOD2 | SLC16A1 | TTR | SCN1A | HRAS |
| ACADM | COL11A2 | FKBP10 | IKBKG | MMACHC | PMM2 | SLC25A13 | TWIST1 | G6PD | KIT |
| ACADS | COL3A1 | FKRP | IL2RG | MMADHC | PNPLA2 | SLC25A15 | TYR | ETFA | KRAS |
| AGK | COL9A1 | FLNA | INPPL1 | MMUT | POR | SLC25A20 | UCP2 | ETFB | NRAS |
| AGPS | COL9A2 | FLNB | INSR | MOCS1 | POU3F4 | SLC35D1 | USH2A | ETFDH | PTPN11 |
| AKR1C2 | COL9A3 | FMO3 | IVD | MOCS2 | PPARG | SLC37A4 | VHL | GCDH | RAF1 |
| AKR1C4 | COMP | GALE | IYD | MRAS | PPIB | SLC5A5 | WDR34 | GJB2 | RIT1 |
| ALPL | CPS1 | GALK1 | KCNH2 | MTM1 | PPP1CB | SMPD1 | WDR35 | GJB6 | SOS1 |
| ANO5 | CPT1A | GALM | KCNJ1 | MT-RNR1 | PROC | SNAI2 | WT1 | OTOF | SPRED1 |
| APOB | CPT2 | GALNS | KCNJ11 | MYO15A | PROKR2 | SOS2 | ZFPM2 | POU4F3 | GPR143 |
| AQP2 | CRTAP | GALT | KCNQ1 | MYO6 | PRPH2 | SOX10 | ZIC2 | SLC26A4 | SLC22A5 |
| ARG1 | CYBA | GATA2 | KIAA0586 | MYO7A | PTS | SOX9 | DDC | F8 | FGF23 |
| ARSA | CYBB | GBA | KIF21A | NAGLU | QDPR | SPG11 | ABCD1 | F9 | PHEX |
| ARSB | CYP11A1 | GCH1 | KIF22 | NAGS | RAG 1 | SPG7 | JAG1 | GLA | CDKL5 |
| ARSL | CYP11B2 | GCK | KITLG | NCF1 | RAG2 | SRD5A2 | NOTCH2 | DMD | FOXG1 |
| ASL | CYP17A1 | GCSH | LAMA2 | NCF2 | RASA1 | SRY | APC | NF2 | MECP2 |
| ASPA | CYP21A2 | GHR | LBR | NDP | RASA2 | STAR | PKHD1 | NF1 | CREBBP |
| ASS1 | CYP7B1 | GLUD1 | LDLR | NDUFS2 | RB1 | STAT3 | ATM | GAA | EP300 |
| ATP8B1 | DBT | GNAS | LDLRAP1 | NDUFV2 | REEP1 | STK11 | COL4A3 | G6PC | RUNX2 |
| AVPR2 | DHH | GNE | LEPRE1 (P3H1) | NEK1 | RHO | STS | COL4A4 | SERPING1 | SLC26A2 |
| BCKDHA | DHX37 | GNPAT | LFNG | NEU1 | RP1 | SURF1 | COL4A5 | HEXA | COL2A1 |
| BCKDHB | DLL3 | GNRHR | LIFR | NPC1 | RRAS | TAZ | EYA1 | EXT1 | COL1A1 |
| BLM | DOCK8 | GUSB | LIPA | NPC2 | RS1 | TBX5 | FOXL2 | EXT2 | COL1A2 |
| BMP1 | DUOX2 | HADH | LMNA | NR1H4 | SCN5A | TBX6 | HDAC8 | ATL1 | FGFR2 |
| BSND | DUOXA2 | HBA1 | LZTR1 | OAT | SCO2 | TCIRG1 | NIPBL | SPAST | FGFR3 |
| BTD | DYNC2H1 | HBA2 | MAGED2 | OCRL | SERPINH1 | TG | RAD21 | ANOS1 | FGFR1 |
| CANT1 | DYSF | HES7 | MAP2K1 | PABPN1 | SGCA | TGIF1 | SMC1A | KDM6A | COL11A1 |
| CAPN1 | EBP | HFE | MAP2K2 | PAH | SGCB | TH | SMC3 | KMT2D | TRAPPC2 |
| CAPN3 | EDA | HLCS | MAP3K1 | PAX3 | SGCD | TJP2 | CFTR | HBB | TSC1 |
| CaSR | EDAR | HMBS | MAT1A | PAX6 | SGCE | TMEM38B | CHD7 | HPRT1 | TSC2 |
| CBL | EDN3 | HNF4A | MATN3 | PAX8 | SGCG | TOR1A | NR0B1 | SLURP1 | OTC |
| CBS | EDNRB | HNRNPDL | MCCC1 | PCBD1 | SGSH | TP53 | WFS1 | FBN1 | CYP27B1 |
| CBX2 | ELP1 | HSD17B3 | MCCC2 | PCSK9 | SHH | TP63 | TGFBR1 | FBN2 | IRF6 |
| CEP120 | ERCC6 | HSD3B2 | MCEE | PDHA1 | SHOC2 | TPO | TGFBR2 | ATP7B | WAS |
